# Effect of Vitamin D Supplements on Relapse or Death in a p53-Immunoreactive Subgroup With Digestive Tract Cancer: Post Hoc Analysis of the AMATERASU Randomized Clinical Trial

**DOI:** 10.1001/jamanetworkopen.2023.28886

**Published:** 2023-08-22

**Authors:** Kazuki Kanno, Taisuke Akutsu, Hironori Ohdaira, Yutaka Suzuki, Mitsuyoshi Urashima

**Affiliations:** 1 Division of Molecular Epidemiology, the Jikei University School of Medicine, Tokyo, Japan; 2 Department of Surgery, International University of Health and Welfare Hospital, Tochigi, Japan

## Abstract

**Importance:**

Recent meta-analyses of randomized clinical trials found that daily vitamin D3 supplementation had beneficial effects on cancer mortality, although the results are still controversial.

**Objective:**

To examine whether vitamin D supplementation reduces the risk of relapse or death in a supgroup of patients with digestive tract cancer who were p53 immunoreactive.

**Design, Setting, and Participants:**

This was a post hoc subgroup analysis of the AMATERASU randomized, double-blind, placebo-controlled clinical trial. This trial included patients at a single university hospital in Japan with digestive tract cancers between January 2010 and February 2018 followed up for a median (IQR) of 3.5 (2.5-5.3) years to compare the effects of vitamin D supplementation with placebo and was reported in 2019. Patients from among 417 participants in the AMATERASU trial whose residual serum samples were available were included. Data were analyzed from October 20 to November 24, 2022.

**Interventions:**

Vitamin D3 (2000 IU/d) supplementation or placebo.

**Main Outcomes and Measures:**

The primary outcome was 5-year relapse or death. The subgroup of patients who were p53 immunoreactive was defined by positivity for anti-p53 antibodies in serum and nuclear accumulation of p53 oncosuppressor protein in more than 99% of cancer cells, which is considered a biomarker for *p53* missense mutations. Anti-p53 antibody levels were measured using chemiluminescent enzyme immune assay. Immunohistochemical staining data of p53 protein in cancer tissue in pathologic specimens were obtained from a previous study and divided into 4 grades.

**Results:**

Among 392 patients with digestive tract cancer (mean [SD] age, 66 [10.7] years; 260 males [66.3%]), there were 37 patients with esophageal cancer (9.4%), 170 patients with gastric cancer (43.4%), 2 patients with small bowel cancer (0.5%), and 183 patients with colorectal cancer (46.7%). Serum anti-p53 antibody was detectable in 142 patients (36.2%), and p53-immunohistochemistry grade showed a positive association with serum anti-p53 antibody levels (coefficient = 0.19; *P* < *.001*). In the p53-immunoreactive subgroup (80 patients), relapse or death occurred in 9 of 54 patients (16.7%) in the vitamin D group and 14 of 26 patients (53.8%) in the placebo group; 5-year relapse-free survival (RFS) was significantly higher in the vitamin D group (13 patients [80.9%]) than the placebo group (1 patient [30.6%]; hazard ratio [HR], 0.27; 95% CI, 0.11-0.61; *P* = .002). This was significantly different from 272 patients in the non–p53 immunoreactive subgroup, in which vitamin D had no effect on 5-year RFS (vitamin D: 35 of 158 patients [22.2%] vs placebo: 24 of 114 patients [21.1%]; HR, 1.09; 95% CI, 0.65-1.84) (*P* for interaction = .005).

**Conclusions and Relevance:**

This study found that vitamin D supplementation reduced the risk of relapse or death in the subgroup of patients with digestive tract cancer who were p53 immunoreactive.

**Trial Registration:**

Identifier: UMIN000001977

## Key Points

**Question:**

Can vitamin D supplementation reduce the risk of relapse or death in a subgroup of patients with digestive tract cancer who were p53 immunoreactive, defined by positivity for anti-p53 antibodies in serum and p53 protein in more than 99% of cancer cells?

**Findings:**

In this post hoc analysis of a randomized clinical trial including 392 patients with digestive tract cancer, 5-year relapse-free survival was significantly higher in the vitamin D group (80.9%) than the placebo group (30.6%) among patients in the p53-immunoreactive subgroup but not in the non–p53-immunoreactive subgroup.

**Meaning:**

These findings suggest that vitamin D supplementation may reduce the risk of relapse or death in this subgroup of patients.

## Teaser

This post hoc analysis of a randomized clinical trial investigates the effect of vitamin D supplementation on risk of relapse or death in a subgroup of patients with digestive tract cancer who were p53 immunoreactive.

## Introduction

Worldwide, an estimated 10.0 million cancer deaths occurred in 2020,^1^ and this number is projected to reach 16.3 million deaths by 2040.^2^ Faced with this alarming trend, researchers have intensified their efforts to identify interventions that can effectively reduce all kinds of cancer mortality. Notably, in the Vitamin D and Omega-3 Trial (VITAL) study,^3^ 2000 IU of vitamin D3 administered daily reduced all cancer mortality by 25% when the first 2 years of observation were excluded. Moreover, a 2023 meta-analysis^4^ of randomized clinical trials (RCTs) found that daily vitamin D3 supplementation was associated with improved cancer mortality in the general population and survival in patients with cancer, although the results are still controversial.^5^ Beneficial effects of vitamin D have been reported for cancers at various sites, and *p53* oncosuppressor is the most frequently mutated gene in approximately half of cancers and relatively common across cancers at all primary sites. These findings suggest that p53 and its related molecules may be plausible targets of vitamin D’s anticancer effects.^6,7^ Indeed, in our previous post hoc study of the AMATERASU RCT, which enrolled patients with digestive tract cancer,^8^ vitamin D supplementation was found to improve relapse-free survival (RFS) in a patient subgroup with nuclear accumulation of p53 oncosuppressor protein, as seen by immunohistochemistry (IHC) staining of pathological specimens.^9^ Strong positivity in the IHC staining was reported as a convenient biomarker for detecting p53 missense mutations with high sensitivity and specificity.^10,11^ Additionally, mutant p53 proteins that accumulated in cancer cells were shown to be immunogenic, leading to generation of anti-p53 antibody (p53-Ab) in vitro^12,13,14^ and have previously been detected in the serum of patients with a variety of cancers.^15,16,17,18,19^ It is assumed that the immune system responds to the abnormal p53 protein as a cancer-specific antigen.

Vitamin D was previously demonstrated to upregulate innate and adaptive immunity.^20,21^ A meta-analysis^22^ of RCTs showed that vitamin D supplementation reduced the incidence of acute respiratory tract infection compared with placebo. In addition, vitamin D supplementation reduced the risk of relapse in the subgroup of patients who had sufficient infiltration of immune cells into the tumor microenvironment in our previous research.^23^ We therefore hypothesized that vitamin D supplements may reduce the risk of relapse or death by enhancing anticancer immunoreaction, indicated by the presence of antibodies against mutated p53 proteins as cancer-specific antigens that are distinct from normal p53. In this post hoc analysis of the AMATERASU RCT,^8^ we explored whether serum levels of p53-Ab were higher in patients with abnormally expressed p53 protein in cancer tissue than those without abnormal expression. We also aimed to examine whether vitamin D supplementation reduced the risk of relapse or death in the subgroup of patients who were p53 immunoreactive, defined by positivity for p53 protein in cancer cells and p53-Ab in serum.

## Methods

The trial protocol (Supplement 1) for this post hoc analysis of an RCT was approved by the ethics committee of the International University of Health and Welfare Hospital, as well as the Jikei University School of Medicine (Nishi-shimbashi, Tokyo, Japan). Written informed consent was obtained from each participating patient. The ethics committee and consent statements apply to the original and current study. This study followed the Consolidated Standards of Reporting Trials (CONSORT) reporting guideline.

### Trial Design

This was a post hoc analysis of parent trial participants from the AMATERASU RCT^8^ focusing on a subgroup of patients who were positive for p53-Ab in serum and p53 protein in pathological specimens. Briefly, the parent trial enrolled 417 patients with stage I to III cancer of the digestive tract from the esophagus to the rectum who underwent curative surgery and participated in a randomized, double-blind, placebo-controlled clinical trial to compare the effects of postoperative daily oral vitamin D3 supplementation (2000 IU/d) vs placebo on relapse or death. Participants were allocated at a ratio of 3:2, and the study was conducted at the International University of Health and Welfare Hospital (Otawara, Tochigi, Japan) between January 2010 and February 2018. Participants were asked to continue supplements until the end of the trial.

### Participants

Of 417 patients with digestive tract cancers who were randomly assigned to receive vitamin D supplements (251 patients [60.2%]) or placebo (166 patients [39.8%]) and who were followed up for a median (IQR; maximum) period of 3.5 (2.5-5.3; 7.6) years, 392 participants (94.0%) had available serum samples and were eligible for this post hoc analysis. Serum samples were not obtained for 25 patients (6.0%). Details of inclusion and exclusion criteria are described in the original report.^8^

### Outcome

The outcome was prespecified as relapse or all-cause death. Elapsed time was defined as the time from starting the supplement to the date the outcome occurred or the patient was censored.

### Detection of p53 Protein in Pathological Specimens

Immunohistochemical staining data of p53 in cancer tissue in pathology specimens was obtained from our previous study.^9^ Briefly, patients were divided into 4 grades based on p53 protein expression patterns in pathological specimens by IHC: (1) overexpressed p53 (p53-IHC [3+]): more than 99% of cellular nuclei in the cancerous region overexpressed p53 protein; (2) strongly expressed p53 (p53-IHC [2+]): some (>10% and ≤99%) cellular nuclei in the cancerous region showed strong p53 positivity; (3) faintly expressed p53 (p53-IHC [1+]): some cellular nuclei in the cancerous region showed faint p53 positivity, and a few cells (≥1% and ≤10%) showed strong p53 nuclear accumulation; (4) p53 not expressed (p53-IHC [0]): strong or faint p53 cells were rarely found in the cancerous region (<1%). To conduct a sensitivity analysis, the study population was also divided into 2 subgroups: with more than 10% (p53-IHC [+]) and 10% or less (p53-IHC [−]) of cellular nuclei in the cancer showing any p53 positivity. The cutoff point of 10% was used in this study because this was found to be the most commonly chosen cutoff point in a meta-analysis of 36 studies on p53 IHC for *p53* gene mutations.^10,11^ Details are described in the original report.^9^

### Serum Anti-p53 Antibody Level Measurement

Patient serum p53-Ab levels at approximately 2 weeks after operation and just before starting trial vitamin D supplementation (ie, at randomization), were measured by SRL, Inc (Shinjuku, Tokyo, Japan) using chemiluminescent enzyme immune assay (CLEIA). The lowest measurable serum p53-Ab level is 0.4 U/mL. The study population was divided into 2 subgroups based on serum p53-Ab levels, with patients having levels of 0.4 U/mL or higher designated as p53-Ab (+) and those having levels less than 0.4 U/mL designated as p53-Ab (−).

### Statistical Analysis

Patients who underwent randomization and for whom residual serum samples were available for measurement of p53-Ab levels were included in this analysis. Relapse and death-related outcomes were assessed according to randomization group (ie, whether supplements were taken) as an intention-to-treat analysis. Effects of vitamin D and placebo on the risk of relapse or death were estimated using Nelson-Aalen cumulative hazard curves. A Cox proportional hazards model was used to determine hazard ratios (HRs) and 95% CIs. The p53-immunoreactive subgroup included patients who were p53-Ab (+) in serum and p53-IHC [3+] in cancer cells. To clarify whether vitamin D supplementation affected outcomes in the p53-immunoreactive subgroup but not in the non–p53-immunoreactive subgroup, *P* values for the interaction were analyzed based on a Cox regression model. The model included treatment allocation, the p53-immunoreactive subgroup, and both factors multiplied together as an interaction variable and analyzed the p53-immunoreactive group by 2-way interaction tests compared with the non-p53-immunoreactive subgroup. Because the *P* value was assessed 5 times, a 2-sided *P* < .01 was considered statistically significant with application of the Bonferroni correction (ie, .05/5 = .01, considering the potential for a type I error due to multiple comparisons). Data were analyzed using Stata statistical software version 17.0 from October 20 to November 24, 2022.

## Results

### Study Population

**Figure 1.**
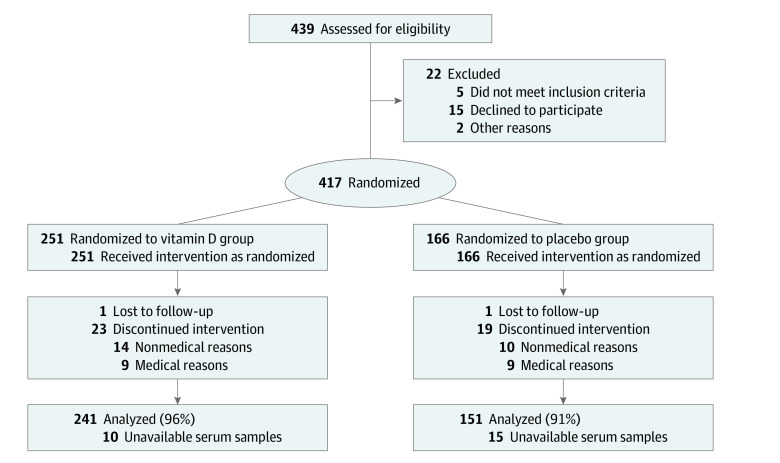
Study Flowchart

The study population consisted of 392 patients with levels of p53-Ab measured (260 males [66.3%]; mean [SD; range] age, 66 [10.7; 35-90] years), including 241 patients in the vitamin D group and 151 patients in the placebo group (Figure 1). The distribution of cancer types and stages were as follows: 37 patients with esophageal cancer (9.4%), 170 patients with gastric cancer (43.4%), 2 patients with small bowel cancer (0.5%), 183 patients with colorectal cancer (46.7%), and 173 patients with stage I (44.1%), 104 patients with stage II (26.5%), and 115 patients with stage III (29.4%) cancer. There were 40 patients (10.2%) with missing histopathology specimens.

### Patient Characteristics

There was detectable p53-Ab in the serum of 142 patients (36.2%), constituting the p53-Ab (+) group, with levels ranging from 0.4 to 1890.0 U/mL, but not in the remaining 250 patients (63.8%), constituting the p53-Ab (−) group. Characteristics of participants in these groups are shown in the Table. Patients in the p53-Ab (+) group more frequently had squamous cell cancer (21 patients [14.8%] vs 14 patients [5.6%]) and less frequently had adenocarcinoma (120 patients [84.5%] vs 232 patients [92.8%]) compared with those in the p53-Ab (−) group. Other characteristics, including intervention group, serum 25-hydroxy vitamin D level, sex, age, and body mass index (calculated as weight in kilograms divided by height in meters squared), were not different between groups.

**Table.** Patient Characteristics

Characteristic	Patients, No. (%) (N = 392)	
	p53-Ab (+) (n = 142)^a^	p53-Ab (−) (n = 250)^b^
Intervention		
Vitamin D	86 (60.6)	155 (62.0)
Placebo	56 (39.4)	95 (38.0)
25(OH)D serum level, ng/mL		
Low: <20	56 (39.4)	105 (42.0)
Middle: ≥20 and ≤40	82 (57.7)	141 (56.4)
High: >40	3 (2.1)	2 (0.8)
Median (IQR)	21 (16-26)	21 (16-28)
Sex		
Male	96 (67.6)	164 (65.6)
Female	46 (32.4)	86 (34.4)
Age quartile, y		
Q1, 35-59	40 (28.2)	58 (23.2)
Q2, 60-65	27 (19.0)	61 (24.4)
Q3, 66-73	34 (23.9)	68 (27.2)
Q4, 74-90	41 (28.9)	63 (25.2)
BMI quartile		
Q1, 15.0-19.7	37 (26.1)	56 (22.4)
Q2, 19.8-21.8	36 (25.4)	63 (25.2)
Q3, 21.9-23.7	36 (25.4)	64 (25.6)
Q4, 23.8-37.3	32 (22.5)	65 (26.0)
History of other cancer	5 (3.5)	11 (4.4)
Comorbidity		
Hypertension	46 (32.4)	105 (42.0)
Diabetes	25 (17.6)	38 (15.2)
Endocrine disease	6 (4.2)	42 (16.8)
Cardiovascular disease	9 (6.3)	20 (8.0)
Chronic kidney disease	5 (3.5)	0
Asthma	1 (0.7)	2 (0.8)
Orthopedic disease	0	2 (0.8)
Site of cancer		
Esophageal	21 (14.8)	16 (6.4)
Stomach	52 (36.6)	118 (47.2)
Small bowel	0	2 (0.8)
Colorectal	69 (48.6)	114 (45.6)
Stage		
I	51 (35.9)	122 (48.8)
II	44 (31.0)	60 (24.0)
III	47 (33.1)	68 (27.2)
Pathology		
Adenocarcinoma	120 (84.5)	232 (92.8)
Squamous cell carcinoma	21 (14.8)	14 (5.6)
Other	1 (0.7)	4 (1.6)
Adjuvant chemotherapy	59 (41.5)	78 (31.2)

Abbreviations: 25(OH)D, 25-hydroxy vitamin D; BMI, body mass index (calculated as weight in kilograms divided by height in meters squared); p53-Ab, anti-p53 antibody.

^a^ ≥0.4 U/mL.

^b^ <0.4 U/mL.

Characteristics of patients who were p53-Ab (+) stratified by intervention are shown in the eTable in Supplement 2. The vitamin D group included more older patients and patients with cardiovascular diseases than the placebo group. Other characteristics did not differ between groups.

### Subgroup Analysis of Patients With and Without p53-Ab in Serum

**Figure 2.**
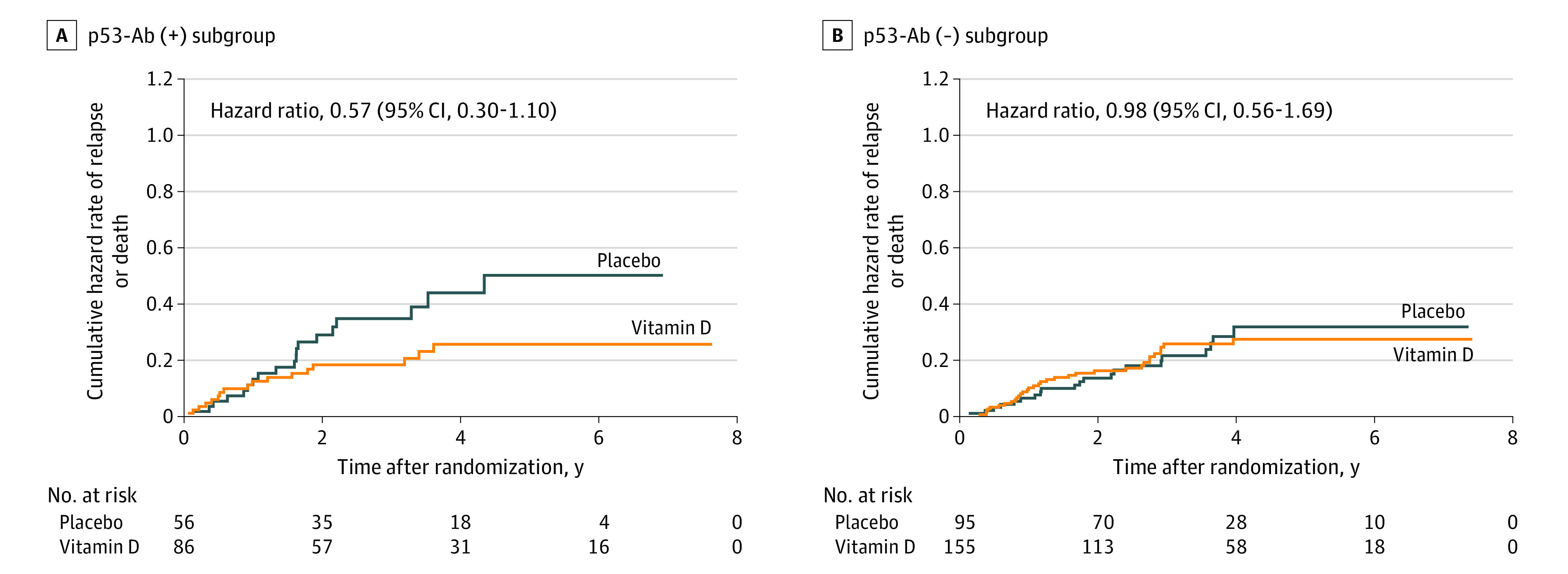
Subgroup Analysis of Patients With and Without Serum Anti-p53 Antibody (p53-Ab) Nelson-Aalen cumulative hazard curves for relapse or death in p53-Ab (+) and p53-Ab (−) groups, assessing patients taking vitamin D vs those taking placebo, were compared using hazard ratios and 95% CIs.

In the p53-Ab (+) group, relapse or death occurred in 17 of 86 patients (19.8%) in the vitamin D group and 19 of 56 patients (33.9%) in the placebo group; 5-year RFS was 26 patients (77.2%) in the vitamin D group and 10 patients (60.0%) in the placebo group, indicating no significant difference (HR, 0.57; 95% CI, 0.30-1.10) (Figure 2A). The difference was significant when adjusted for age and a history of cardiovascular diseases (HR, 0.47; 95% CI, 0.23-0.95). In the p53-Ab (−) group, relapse or death occurred in 33 of 155 patients (21.3%) in the vitamin D group and 21 of 95 patients (22.1%) in the placebo group; the 5-year RFS was 37 patients (75.9%) in the vitamin D group and 20 patients (72.5%) in the placebo group, which were not significantly different (HR, 0.98; 95% CI, 0.56-1.69) (Figure 2B), with no 2-way interaction between vitamin D supplementation and the subgroup of patients who were p53-Ab (+).

### Association Between Expression of p53 Protein and Serum p53-Ab Levels

**Figure 3.**
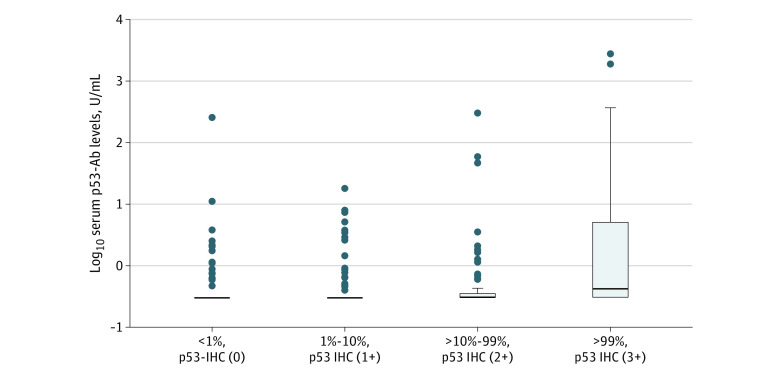
Serum Anti-p53 Antibody (p53-Ab) Levels by p53 Immunohistochemistry (IHC) Protein Expression IHC levels were 0 (<1% strong or faint p53 cells in cancerous region; p53-IHC [0]), 1 (1%-10% strong p53 cells in cancerous region; p53 IHC [1+]), 2 (>10%-99% strong p53 cells in cancerous region; p53 IHC [2+]), and 3 (>99% overexpressed p53 cells in cancerous region; p53 IHC [3+]).

Figure 3 shows log-transformed serum p53-Ab levels according to p53 protein expression (ie, p53-IHC [0], p53-IHC [1+], p53-IHC [2+], and p53-IHC [3+]). There was a positive association between p53-IHC grades and serum p53-Ab levels in linear regression analysis (coefficient = 0.19; *P* < *.001*). Similarly, mean (SD) serum p53-Ab levels were significantly higher in patients who were p53-IHC (+) (32.9 [227.1] U/mL) than in those who were p53-IHC (−) (2.7 [21.5] U/mL) (Mann-Whitney test; *P* < .0001).

### Analyses in p53-Immunoreactive Subgroup

**Figure 4.**
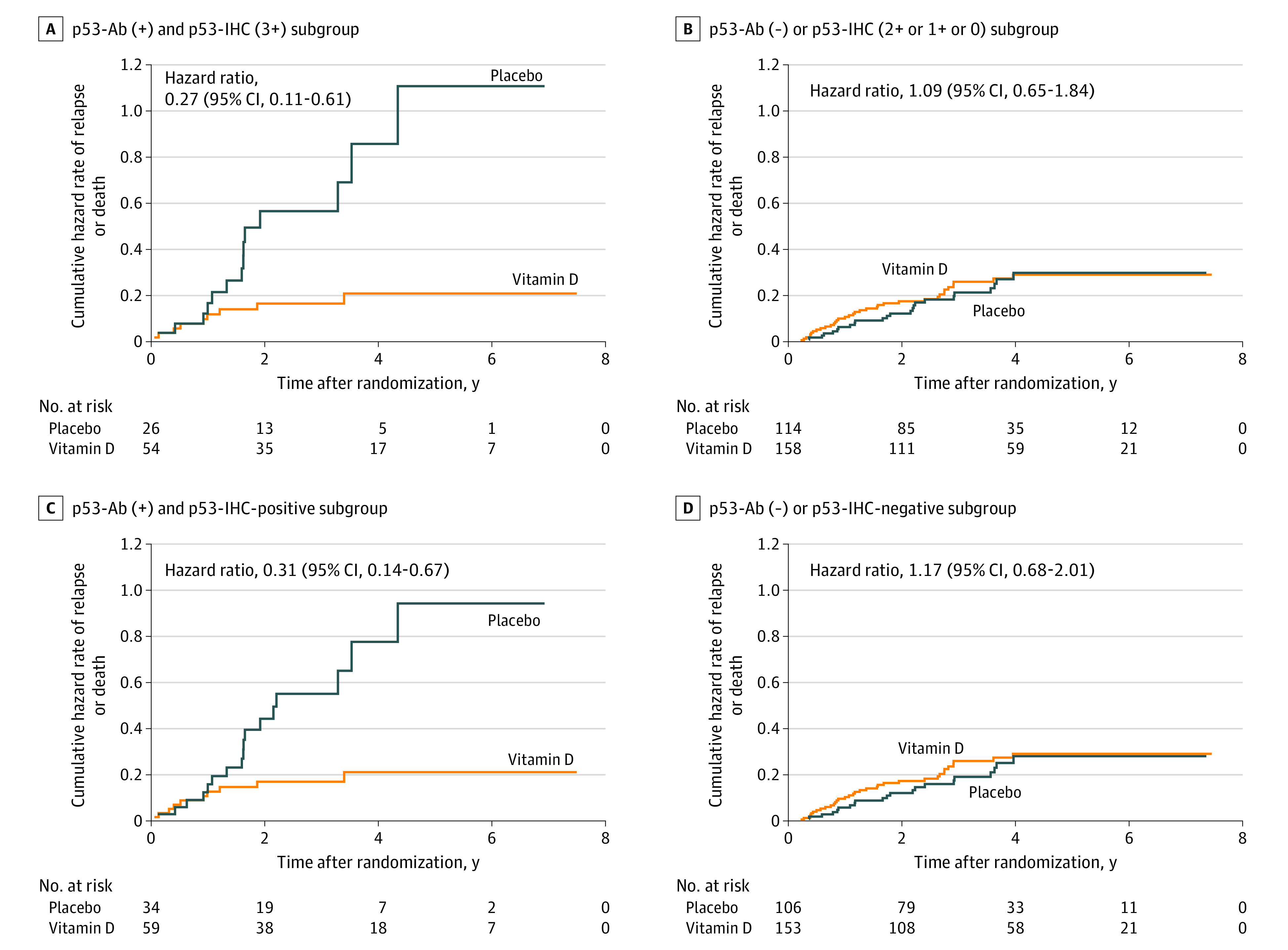
Analysis of p53-Immunoreactive Subgroup IHC indicates immunohistochemistry; p53-Ab, anti-p53 antibody. Nelson-Aalen cumulative hazard curves are presented in the p53-Ab (+) and p53-IHC (3+) subgroup, p53-Ab (−) or p53-IHC (2+, 1+, or 0) subgroup, p53-Ab (+) and p53-IHC-positive subgroup, and p53-Ab (−) or p53-IHC-negative subgroup. Hazard curves for relapse or death in patients taking vitamin D were compared with those taking placebo.

In 80 patients in the p53-immunoreactive subgroup (ie, patients who were p53-Ab [+] and p53-IHC [3+]), relapse or death occurred in 9 of 54 patients (16.7%) in the vitamin D group and 14 of 26 patients (53.8%) in the placebo group; the 5-year RFS was significantly higher in the vitamin D group (13 patients [80.9%]) than the placebo group (1 patient [30.6%]; HR, 0.27; 95% CI, 0.11-0.61; *P* = .002; estimated power > 0.99) (Figure 4A). The difference remained significant after adjustment for age and history of cardiovascular disease (HR, 0.20; 95% CI, 0.08-0.50). In 272 patients in the non–p53-immunoreactive group (ie, patients who were p53-Ab [−] or p53-IHC [2+, 1+, or 0] negative), relapse or death occurred in 35 of 158 patients (22.2%) in the vitamin D group and 24 of 114 patients (21.1%) in the placebo group; the 5-year RFS was 37 patients (74.7%) in the vitamin D group and 24 patients (74.1%) in the placebo group, indicating no significant difference (HR, 1.09; 95% CI, 0.65-1.84) (Figure 4B), which was significantly different from the effect of vitamin D in the p53-immunoreactive subgroup (*P* for interaction = 0.005).

To conduct a sensitivity analysis using a cutoff p53-IHC value of 10% instead of 99%, patients were divided into 2 groups of p53-IHC (+), or greater than 10%, and p53-IHC (−), or 10% or less. In the p53-Ab (+) and p53-IHC (+) group, relapse or death occurred in 10 of 59 patients (16.9%) in the vitamin D group and 17 of 34 patients (50.0%) in the placebo group; the 5-year RFS was significantly higher in the vitamin D group (14 patients [80.7%]) than the placebo group (2 patients [37.4%]; HR, 0.31; 95% CI, 0.14-0.67; estimated power > 0.99) (Figure 4C). The difference remained significant after adjustment for age and history of cardiovascular disease (HR, 0.24; 95% CI, 0.10-0.57). In the p53-Ab (−) or p53-IHC (−) group, relapse or death occurred in 34 of 153 patients (22.2%) in the vitamin D group and 21 of 106 patients (19.8%) in the placebo group; the 5-year RFS was 36 patients (74.6%) in the vitamin D group and 23 patients (75.4%) in the placebo group, indicating no significant difference (HR, 1.17; 95% CI, 0.68-2.01) (Figure 4D). This was significantly different from the effect of vitamin D in the p53-immunoreactive subgroup (*P* for interaction = .006).

## Discussion

In this post hoc analysis of the AMATERASU RCT, p53-IHC grades showed a positive association with serum p53-Ab levels. Additionally, p53-Ab levels were significantly higher in patients who were p53-IHC (+) than those who were p53-IHC (−). Previously, mutant p53 protein that accumulated in cancer cells was reported to be immunogenic and to lead to the generation of p53-Ab in vitro.^12,13,14^ These results suggest the possibility that patient immune systems may recognize the abnormally expressed p53 protein as a cancer-specific antigen.

The main findings of this study were that daily supplementation of 2000 IU of vitamin D reduced the risk of relapse or death by 27% compared with placebo in the p53-immunoreactive subgroup, defined by positivity for p53-Ab in serum and p53 protein in more than 99% of cancer cells. Results indicated interactions between vitamin D supplementation and p53-immunoreactive subgroup even after Bonferroni correction. Moreover, results showed a similar risk reduction by shifting the cutoff point of p53-IHC from more than 99% to 10% for sensitivity analysis. As preliminary evidence, we previously reported that vitamin D reduced the risk of relapse or death by 52% compared with the placebo group in the p53-IHC (+) cancer subgroup in a post hoc analysis of the same AMATERASU trial.^9^ Building on the previous study, this study’s results additionally suggest that vitamin D supplementation may be more effective in improving prognosis in patients who are p53-Ab (+) in serum and p53-IHC (+) in cancer tissue compared with patients who are only p53-IHC (+) independent of p53-Ab status. Another previous study of a murine model^24^ showed that p53-Ab treatment could prevent metastasis of colon cancer cells to the liver. Although further research is needed, these results suggest that cancer immunotherapy targeting mutated p53 proteins should be developed.

### Limitations

This study has several limitations. First, this was a post hoc analysis of the AMATERASU RCT, and the number of patients in the p53-immunoreactive subgroup was very small. Second, because analyses assessed a post hoc hypothesis, observer error or bias could have influenced results. Thus, the findings must be considered exploratory and interpreted with caution, although the main results of this study were remarkable and remained significant even after Bonferroni correction. Third, mutations of the *p53* gene were not directly sequenced. Thus, observation of abnormal expression of the p53 protein by IHC may not always imply mutations in the *p53* gene. Fourth, because serum levels of p53-Ab were measured at the first outpatient visit at approximately 2 weeks after tumor resection by operation, their values may have been lower than before the operation. Fifth, the number of patients analyzed was lower than in the original study because serum samples were not obtained for 6.0% of them. However, patient characteristics in this post hoc study did not substantially differ from those in the original trial. Sixth, because the AMATERASU trial was conducted in Japan, all patients were Asian, most esophageal cancers were squamous cell carcinomas, the incidence of gastric cancer was relatively high, and the bioavailability of 25-hydroxy vitamin D could be different from that in other population groups.^25,26^ Seventh, 40 of 392 patients (10.2%) in this study had missing histopathology specimens, which may have biased results of analyses toward the null hypothesis. Eighth, it is uncertain in digestive tract cancers whether squamous cell carcinoma and adenocarcinoma with different characteristics can be analyzed together. Thus, results of this study may not necessarily be generalizable to other populations.

## Conclusions

This post hoc analysis of an RCT found that vitamin D supplementation reduced the risk of relapse or death in the subgroup of patients with digestive tract cancer who were p53 immunoreactive. Findings suggest the importance of developing cancer immunotherapy targeting mutated p53 proteins.

## Notes

Supplement 1.
Trial Protocol and Statistical Analysis Plan

Supplement 2. **eTable.** Characteristics of Patients Who Were Anti-p53 Antibody Positive by Intervention

Supplement 3.
Data Sharing Statement
