# Supplementary material for: Effect of Vitamin D Supplements on Relapse or Death in a p53-Immunoreactive Subgroup With Digestive Tract Cancer: Post Hoc Analysis of the AMATERASU Randomized Clinical Trial
Source: JAMA Netw Open. 2023 Aug 22;6(8):e2328886. doi: 10.1001/jamanetworkopen.2023.28886 (PMC10445201; doi:10.1001/jamanetworkopen.2023.28886)
Supplement: Supplement 1. — Trial Protocol and Statistical Analysis Plan [file jamanetwopen-e2328886-s001.pdf]

Trial Protocol and Statistical Analysis Plan (SAP)

Initial Trial Protocol and Statistical Analysis Plan (SAP) in English p2 ~ p16

Final Trial Protocol and Statistical Analysis Plan (SAP) in English p17 ~ p33

References p34 ~ p35

Appendix 1: Table 1. Primers and PCR conditions p36

Appendix 2: Table 2. Permutated blocks of five p37

Appendix 3: Table 2. Permutated blocks of five p38 ~ p44

Summary of changes to the protocol approved by the ethics committee p45

***The statistical analysis plan is contained within the protocol files.**

Ver. 1. 2008.12.25

Trial Protocol and Statistical Analysis Plan (SAP) (Initial)

Title: A randomized, double blind, comparative study of vitamin D3 versus placebo in patients with cancer in gastrointestinal tract to prevent relapse after operation

Steering committee

Lead principal investigator
Mitsuyoshi Urashima MD, PhD, MPH
Professor of Molecular Epidemiology
The Jikei University School of Medicine
Tokyo, JAPAN

Co-investigator

Yutaka Suzuki MD, PhD
Professor of Surgery
International University of Health and Welfare,
Tochigi, JAPAN

Hironori Ohdaira MD, PhD
Associate Professor of Surgery
International University of Health and Welfare,
Tochigi, JAPAN

Protocol synopsis

Background

1. The prognoses of patients with colorectal cancer, and lung cancer, and other cancers are poorer for those with lower serum levels of vitamin D.
2. Among patients with lung cancer and Fok*I* polymorphisms of vitamin D receptor (VDR), the median survival periods for patients with CC, CT, and TT genotypes are 21.4, 12.1, and 15.6 months, suggesting that polymorphisms of VDR significantly impact prognosis (log rank P = 0.005).

Objectives

1. To determine whether vitamin D3 (1,200 IU) can prevent relapse and death after surgical treatment for patients with gastrointestinal tract (esophageal gastric and colon) cancer compared with a placebo.
2. To determine relationships between relapse-free survival and serum vitamin D concentrations, SNPs of the vitamin D receptor (VDR) and vitamin D binding protein (DBP) in patients with cancers of the gastrointestinal tract (esophageal, gastric, and colon cancer).

Study design

Randomized, double blind, placebo-controlled, parallel two-group trial, with serum concentration of vitamin D, that is 25(OH)D, and SNP analysis

Participants

Patients with cancers of the gastrointestinal tract: Total, n = 400; vitamin D group, n = 240; placebo group, n = 160.

Location

a. Patient care: The International University of Health and Welfare (IUHW) Hospital
b. Data monitoring and analysis: Division of Molecular Epidemiology, the Jikei University School of Medicine (JUSM) (data monitoring and analysis)

Study period

Accrual, if the number of randomized patients reaches >400, then entry is stopped.
The trial ends if patient around ID400 is followed up for 2 years.

Methods

1. When patients are considered eligible, the collaborating surgeons describe the trial to the patients and their families at the hospital outpatient clinic or upon admission, and seek their agreement to participate. Written, informed consent (IC) obtained from each participant is then sent with an entry document to the Division of Molecular Epidemiology at JUSM. All personal information about the participants was rendered innominate and changed to a study ID.
2. Before starting supplements, blood is sampled and sent to SRL (outsource laboratory) to measure serum 25(OH)D levels and purify genomic DNA.
3. SNPs of VDR and DBP are analyzed by PCR and direct sequencing at the Division of Molecular Epidemiology at JUSM.
4. The enrolled patients are randomly assigned to receive either vitamin D3 or a placebo at the Division of Molecular Epidemiology at JUSM. At IUHW Hospital, the patients are instructed to swallow two capsules per day (containing either supplement or placebo) until the end of the study. Outpatients receive capsules from the CRC.
5. The CRC informs the Division of Molecular Epidemiology at JUSM of the study endpoint by fax.

Outcomes

Primary outcome: relapse-free survival (RFS)

Secondary outcome: overall survival (OS), relapse, all-cause death, cancer-specific death

Safety outcomes: kidney stone, bone fracture, serious adverse events requiring admission. Cancer that appears *de novo* in organs other than the site of the primary cancer after starting supplementation will be included as an adverse event, not as an outcome.

Statistical analysis

Intent to treat, Kaplan-Meier survival curves, Cox hazards models.

Ethics

1. Private information is carefully protected, since human genomes are analyzed.
2. All personal information about the participants is rendered innominate in a linkable fashion at IUHW Hospital.
3. Liability insurance is obtained to compensate patients for side effects of vitamin D or the placebo.

Funding

This study receives funding from the Department of Surgery, IUHW Hospital, Division of Molecular Epidemiology at JUSM and the Ministry of Education, Culture, Sports, Science and Technology in the Japan-Supported Program for the Strategic Research Foundation at Private Universities

Background

Higher serum vitamin D3: 25(OH)D levels are associated with longer survival. The prognosis is poorer for patients with colorectal and lung cancer accompanied by lower, than higher serum levels of vitamin D, which we also confirmed in colorectal cancer.

Randomized controlled trials (RCT) have investigated whether vitamin D plus calcium supplementation can decrease cancer incidence, but none have aimed to improve the prognosis of patients with cancer. Two major RCT have also investigated whether vitamin D and calcium can prevent fracture as a primary outcome and reduce the incidence of cancer as a secondary outcome. However, whether vitamin D supplementation can improve the survival of patients with cancer has not been investigated in an RCT as far as we can ascertain.

Therefore, we planned the first randomized, double-blind, placebo-controlled trial to clarify whether vitamin D3 supplementation can improve relapse-free survival (RFS) and overall survival (OS) among all patients and subgroups of patients with digestive tract cancers from the esophagus to the rectum after curative surgical tumor resection.

Median survival durations of 21.4, 12.1, and 15.6 months are associated with CC, CT, and TT genotypes among Fok*I* SNPs of the vitamin D receptor (VDR) in patients with lung cancer. These findings suggest that SNPs of VDR may significantly impact prognosis (log rank $P = 0.005$).

We therefore planned to determine relationships between relapse-free survival (RFS) and serum 25(OH)D levels, and SNPs of VDR in patients with cancers of the gastrointestinal tract (esophageal, gastric, and colon cancers).

Hypothesis

Mechanisms of anti-cancer effects by vitamin D

Serum levels of the active vitamin D precursor, 25(OH)D increase in response to exposure to sunlight or a vitamin D-rich diet or vitamin D supplementation. In contrast, levels of 1,25$(OH)_2$D that is activated in the kidneys remain constant and are least affected by lifestyle. Cancer cells expressing both 1α-hydroxylase and vitamin D receptor (VDR) uptake 25(OH)D and convert it into 1,25$(OH)_2$D, which binds to VDR in cancer-cell nuclei. This

signaling influences gene expression, which consequently induces cell proliferation and apoptosis, and inhibits proliferation, angiogenesis, and metastasis.

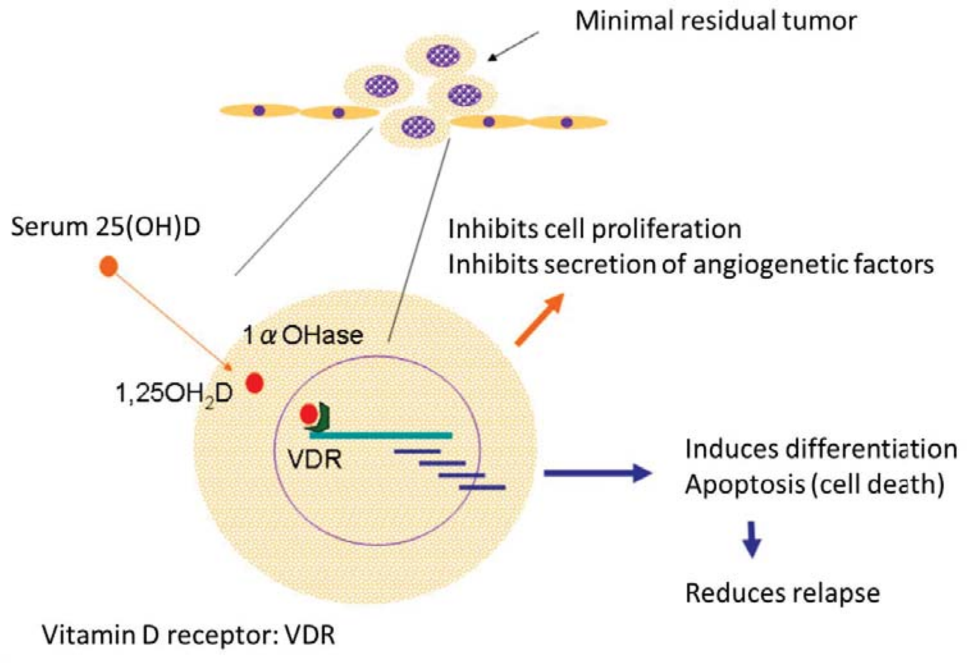

1. Oral vitamin D supplementation causes serum levels of vitamin D (25(OH)D) to increase.
2. Minimal postoperative residual tumors uptake serum 25(OH)D into cancer cells and convert it into active vitamin D (1,25(OH)2D), which binds to nuclear vitamin D receptors within the same cell and influences various cellular functions.
3. As a result, cell proliferation and angiogenesis are suppressed, differentiation and cell death are induced, and minimal residual tumor disappears.
4. Survival can be prolonged by vitamin D compared with a placebo.

Objectives

Specific Aim 1: To determine whether vitamin D3 (1,200 IU) can prevent relapse and death after surgical treatment for cancers patients with the gastrointestinal tract (esophageal gastric and colon) compared with a placebo.

Specific Aim 2: To determine relationships between relapse-free survival and serum vitamin D concentrations, SNPs of the vitamin D receptor (VDR) in patients with cancers of the gastrointestinal tract (esophageal, gastric, and colon cancer).

Study design

Specific Aim 1

At the International University of Health and Welfare (IUHW) Hospital, patients with digestive tract cancers from the esophagus to the rectum are randomized in a double-blind, placebo-controlled, parallel-group trial of the effects of vitamin D3 supplements (1,200IU/day) compared with those of a placebo at an allocation ratio of 3:2 after surgical tumor resection with intent to cure. Relapse-free survival (RFS) and overall survival (OS) are compared between the two groups.

Specific Aim 2

Relationships between RFS and subgroups of patients with the serum 25(OH)D levels described above and SNPs of VDR are analyzed.

Research Implementation System

The following medical institutions will collaborate in this trial.

I. **Jikei University School of Medicine**

Division of Molecular Epidemiology

a. Urashima M: Conception, design, randomization and data analyses
b. Akutsu T: Data monitoring
c. Wada H: Data monitoring
d. Sakanashi C: SNP analysis
e. Tago N: SNP analysis
f. Mafune H: SNP analysis
g. Suga D: SNP analysis

II. **International University of Health and Welfare Hospital（IUHW Hospital）**

Department of Surgery

a. Suzuki Y: Patient entry, treatment, data collection
b. Ohdaira H: Patient entry, treatment, data collection
c. Yoshida M: Patient entry, treatment, data collection
d. Okada S: Pathology
e. Kitajima M: Critical appraisal of draft
a. Ohtsuki Y: Clinical Research Coordinator (CRC)

Patients

Inclusion criteria

1. Histopathologically diagnosed epidermal carcinoma of the digestive tract (esophageal, gastric, small intestinal or colorectal mucosa).
2. Clinical stages I to III.
3. Age 30 – 90 years at entry.
4. Diagnosed and first surgery at IUHW Hospital.
5. Not taking vitamin D supplement or active vitamin D.
6. No previous history of urinary tract stones.

Exclusion criteria

1. Tumor that could not be totally resected by surgery.
2. Serious postoperative complications before starting supplementation.
3. Pathological diagnosis other than epidermal carcinoma (malignant lymphoma, sarcoma, etc.).
4. Pathological stage 0 or IV.

Interventions

Per oral supplementation with vitamin D3 or placebo at IUHW Hospital

Enrolled patients are randomly assigned to receive either vitamin D3 supplements (2 × 600 IU capsules/day) or placebo (2 capsules/day) starting from the first postoperative assessment as an outpatient until the end of the trial. The two capsules could be taken together or as one each twice daily. The placebo comprised sesame oil, gelatin derived from swine, and glycerin and the active supplement contained the same constituents plus vitamin D3.

Outcome Measures

Primary outcome

1. Relapse-free survival (RFS) is defined as elapsed time from starting supplementation to the earliest date of cancer relapse or death from any cause. Participants who do not relapse and remain alive on the day the trial ends are censored. Survival duration is defined as being from the supplement start day to final outpatient day.

Secondary outcomes

1. Overall survival (OS) defined as elapsed time from the date of starting supplementation to the date of death from any cause. Participants who remain alive on the day the trial ends are

censored. Survival duration is defined as being from the supplement start day to the final outpatient day.

2. Relapse: Patients were periodically (1–6 months) examined by CT, MRI, PET and other modalities as needed on an outpatient basis to exclude cancer relapse.

3. All-cause death.

4. Death due to progressive cancer, excluding *de novo* cancer.

5. Death from non-cancer causes such as myocardial infarction and *de novo* cancer progression.

Safety outcomes

1. Urinary stone.
2. Hypercalcemia.
3. Bone fracture.
4. Serious events requiring admission.

Flow of participants

1. **Informed consent and registration** at IUHW Hospital

   When a patient is considered eligible, the collaborating surgeon describes the trial purposes etc. to the patients and their families at the hospital outpatient clinic or during admission before surgery and seeks their agreement to participate. Written, informed consent is obtained from each participant. The participant is then assigned an identification number for a study ID and a registration form with the study ID, age, sex, and key inclusion and exclusion criteria and without personal information is sent by fax from IUHW Hospital to the data monitoring center at JUSM.

2. **Surgical curative resection of tumor and chemotherapy** at IUHW Hospital

   The following are grounds for excluding patients after initial registration:

1. Tumor that could not be totally resected by surgery.

2. Serious postoperative complications before starting supplementation.

3. Pathological diagnosis other than epidermal carcinoma (malignant lymphoma, sarcoma, etc.).

4. Pathological stage 0 or IV.

Chemotherapy

a. Pre- and post-operative chemotherapy is administered to patients with stage II and III esophageal cancer.

b. Post-operative chemotherapy is administered to patients with stage II and III gastric cancer
c. Post-operative chemotherapy is administered to patients with stage III colorectal cancer.
d. Local radiation or molecular targeting therapy is combined with chemotherapy for selected patients with relapse.

3. **Clinical information before intervention** at IUHW Hospital

The following information is summarized by the CRC and sent to the data monitoring center at JUSM by fax.

1. Age
2. Sex
3. Diagnosis (e.g., gastric cancer)
4. Stage before operation
5. Pathological stage
6. Pathology
7. Tumor resection: complete resection; microscopically not resected (=edge positive); macroscopic residual tumor remained in the body
8. Sampling: serum for 25(OH)D; blood for genomic DNA extraction; tumor tissue for somatic DNA extraction
9. Anthropometric measurements: height, weight, abdominal circumference, blood pressure
10. Blood tests: Calcium, ALP, parathyroid hormone, total cholesterol, HDL-cholesterol, triglyceride, blood sugar, HbA1c, BUN, Cr

4. **Blood sampling** at IUHW hospital

Blood sampled for serum 25(OH)D measurements and DNA extraction at IUHW Hospital is sent to SRL Inc.

A) Measurement of serum 25(OH)D levels

Serum levels of 25(OH)D are measured by radioimmunoassay at SRL Inc. (Hachioji, Tokyo, Japan) before and annually (around the same calendar month) after starting supplementation. Levels for 25(OH)D and residual serum samples are sent to the data monitoring center at JUSM for storage at -80°C for post hoc

analysis.

B) SNP analyses of vitamin D receptor

Peripheral blood are sampled from participants at IUHW hospital and sent to SRL Inc., where DNA is extracted. Purified genomic DNA is sent from SRL Inc. to Division of Molecular Epidemiology at JUSM. DNA fragments are amplified by PCR using the forward/reverse primers listed below and the conditions described in. The SNPs are determined by direct sequencing. Samples are stored at -80°C.

SNPs

a. Vitamin D receptor (VDR): Fok*I*, rs10735810; Bsm*I*, rs1544410; CDX2, rs11568820; Apa*I*, rs7976091; Taq*I*, rs731236

C) DNA extraction from tumor tissue

Tumor samples obtained during surgery at IUHW Hospital are sent to SRL Inc., where DNA is extracted. Purified somatic DNA is sent from SRL Inc. to the data monitoring center at JUSM and stored at -80°C for future studies.

5. **Randomization and double blinding** at JUSM

a. Supplementation

Both vitamin D3 and placebo (Zenyaku Pharmaceutical Co., Ltd., Tokyo, Japan) are prepared as soft capsules containing either 1,000 IU of vitamin D3 or a placebo. All capsules are identical in appearance and taste, and packaged in lots of 366 capsules in identical brown glass bottles. Both supplements are purchasable from Zenyaku Pharmaceutical Co., Ltd., Tokyo, Japan.

b. Randomization

M.U. at the data monitoring center has no clinical involvement in this trial. M.U. generates random numbers from 1 to 10 using a computer, assigns permutated blocks of five to fit in a 3:2 ratio, and creates a correspondence table to link the study ID to either vitamin D3 or placebo.

c. Double-blinding

An administrative staff member and M.U. label each bottle with the study ID and confirm the ID number with the correspondence table. Bottles labeled in this manner are periodically sent from the data monitoring center to IUHW Hospital. Staff at the data monitoring center have no contact with participants at IUHW Hospital. Thus, the

participants in this trial and all the staff including surgeons who assess relapse at IUHW Hospital are completely blinded to which patients received supplement or placebo.

6. **Compliance with supplementation** at IUHW Hospital
    1. Patients are questioned about compliance at every visit.
    2. Levels of 25(OH)D are annually measured in blood samples to determine changes in the vitamin D and placebo groups.

7. **Reports of relapse and death** at IUHW Hospital

    Reports of relapse or death are prepared at IUHW Hospital and sent to the data monitoring center at JUSM by fax.

    a. **Relapse**

    In addition to date of relapse, the surgeon in charge or the CRC describes in detail why a patient is diagnosed as having relapsed from MRI findings, and other findings, such as local recurrence, lymph node metastasis, distant metastasis, or peritoneal dissemination.

    b. **Death**

    In addition to the date of death, the surgeon in charge or the CRC details causes of death, such as cancer progression, to determine death from cancer or non-cancer.

    c. **Censor**

    Participants are censored in terms of RFS if they are relapse-free or alive at the end of the trial.

    Participants are censored in terms of OS if they have not died of any cause at the end of the trial. Survival is defined as elapsed time between the dates of starting supplementation and the final visit to the outpatient clinic.

8. **Reports of safety outcomes** at IUHW Hospital

    Reports of safety outcomes (urinary stone, hypercalcemia, bone fracture, severe adverse events requiring admission, double cancer, and others) prepared at IUHW hospital are sent to the data monitoring center at JUSM by fax.

    If medically considered difficult to continue taking supplements or if a participant desires to stop taking supplements, the surgeon in charge can decide to stop supplementation.

9. **Follow-up** at IUHW hospital

   At least once per year, the CRC reports the date and status of participants at their final visit to the outpatient clinic to personnel at the data management center to ensure that all participants are followed up.

Statistical analysis

1. **Sample size**

2. **Sample size calculation**

3. **Study period**

   Fifty patients each with gastric and colorectal cancers and 10 with esophageal cancer are treated annually at the Department of Surgery, IUHW Hospital. We assumed that 80 patients per year could participate in this trial. Therefore, the accrual period will be 5 years.

4. **Interim analysis**

5. **Planned methods of analysis**
   a. Changes of 25(OH)D levels will be analyzed using Wilcoxon signed-rank tests.
   b. Comparisons of patients' characteristics between vitamin D and placebo will be analyzed using Student t-tests and Mann-Whitney tests for continuous variables with normal and non-normal distribution, respectively. Dichotomous outcomes are calculated using chi-square tests.
   c. Kaplan-Meier survival curves will be created on an intent-to-treat analysis.
   d. Cox proportional hazard model will be used to determine hazard ratio (HR) and 95% confidence intervals (95%CI) of RFS and OS.
   e. Relapse and safety outcomes will be evaluates using risk ratio (RR).
   f. All reported P values will be two-sided.
   g. Values with $P < 0.05$ will be considered statistically significant.
   h. All data will be statistically analyzed using Stata 14.0 (StataCorp LP., College Station, TX, USA).

6. **Subgroup analyses**

   To clarify whether vitamin D supplementation significantly affects the subgroups listed

below, P for interaction ($P_{interaction}$) is computed by creating multiplicative variables. The results of these analyses are not corrected for multiple comparisons.

a. **Subgroup analyses**

VDR: Fok*I*, Bsm*I*, CDX2, Taq*I*, Apa*I*

Safety

Serious adverse events or side effects caused by vitamin D3 supplements are rare.

All capsules contain sesame oil, gelatin derived from swine, and glycerin; thus, participants in the vitamin D and placebo groups who might be sensitive to any of these components could develop nausea and vomiting.

The Japanese Ministry of Health, Labor and Welfare suggests that the safe range of vitamin D3 supplementation is between 200 and 2,000 IU/day for healthy adults.

Early withdrawal

a. Unknown serious adverse events and side effects of vitamin D.
b. Frequent known side effects such as nausea and vomiting.
c. Frequent theoretically plausible side effects such as hypercalcemia.

Compensation

Mitsui Sumitomo Insurance Fire provides liability insurance for the principal investigator, to compensate for costs of treating disability as a result of side effects caused by taking the trial supplement.

The maximum amount of compensation is 100,000,000 yen per person and 300,000,000 yen per trial.

Consideration concerning the protection of human rights and privacy

1. Written informed consent is obtained after sufficient explanation.
2. Participants can withdraw from the trial after providing written, informed consent.
3. Withdrawal is not considered a disadvantage for participants.
4. Private information is exchanged with study ID at IUHW Hospital; therefore, private information cannot leak from IUHW Hospital (linkable anonymizing).

5. Private information is not collected at Division of Molecular Epidemiology, JUSM.
6. Contact point regarding study ID.

Disclosure of genetic information

Information disclosure regarding vitamin D related SNPs can be provided by the surgeon in charge at IUHW Hospital if a participant requests such.

Publication of research results

We aim to publish the results of this trial in a scientific journal or present them at academic conferences. Personal information will be carefully concealed when we present the results.

Research funds

The present study will receive funding from the Department of Surgery, IUHW Hospital, Division of Molecular Epidemiology, the Ministry of Education, Culture, Sports, Science and Technology in the Japan-Supported Program for the Strategic Research Foundation at Private Universities

Attribution of intellectual property rights

If intellectual property rights such as patent rights become relevant, such rights will be attributable to the investigator.

Ver. 1. 2012.12.18

Trial Protocol and Statistical Analysis Plan (SAP) (final)

Title: A randomized, double blind, comparative study of vitamin D3 versus placebo in patients with cancer in gastrointestinal tract to prevent relapse after operation

Steering committee

Lead principal investigator
Mitsuyoshi Urashima MD, PhD, MPH
Professor of Molecular Epidemiology
The Jikei University School of Medicine
Tokyo, JAPAN

Co-investigator

Yutaka Suzuki MD, PhD
Professor of Surgery
International University of Health and Welfare,
Tochigi, JAPAN

Hironori Ohdaira MD, PhD
Associate Professor of Surgery
International University of Health and Welfare,
Tochigi, JAPAN

Protocol synopsis

Background

1. The prognoses of patients with colorectal cancer, lung cancer, and other cancers are poorer for those with lower serum levels of vitamin D.
2. Among patients with lung cancer and Fok*I* polymorphisms of vitamin D receptor (VDR), the median survival periods for patients with CC, CT, and TT genotypes are 21.4, 12.1, and 15.6 months, suggesting that polymorphisms of VDR significantly impact prognosis (log rank P = 0.005).

Objectives

1. To determine whether vitamin D3 (2,000 IU) can prevent relapse and death after surgical treatment for patients with gastrointestinal tract (esophageal gastric and colon) cancer compared with a placebo in total study population and in subgroup stratified by serum 25(OH)D levels with cutoffs at 20 and 40 ng/mL.
2. To determine relationships between relapse-free survival and serum vitamin D concentrations, SNPs of the vitamin D receptor (VDR) and vitamin D binding protein (DBP) in patients with cancers of the gastrointestinal tract (esophageal, gastric, and colon cancer).

Study design

Randomized, double blind, placebo-controlled, parallel two-group trial, with serum concentration of vitamin D, that is 25(OH)D, and SNP analysis

Participants

Patients with cancers of the gastrointestinal tract: Total, n = 400; vitamin D group, n = 240; placebo group, n = 160.

Location

a. Patient care: The International University of Health and Welfare (IUHW) Hospital
b. Data monitoring and analysis: Division of Molecular Epidemiology, the Jikei University School of Medicine (JUSM) (data monitoring and analysis)

Study period

Accrual, if the number of randomized patients reaches >400, then entry is stopped.
The trial ends if patient ID400 is followed up for 2 years.

Methods

1. When patients are considered eligible, the collaborating surgeons describe the trial to the patients and their families at the hospital outpatient clinic or upon admission, and seek their agreement to participate. Written, informed consent (IC) obtained from each participant is then sent with an entry document to the Division of Molecular Epidemiology at JUSM. All personal information about the participants was rendered innominate and changed to a study ID.
2. Before starting supplements, blood is sampled and sent to SRL (outsource laboratory) to measure serum 25(OH)D levels and purify genomic DNA.
3. SNPs of VDR and DBP are analyzed by PCR and direct sequencing at the Division of Molecular Epidemiology at JUSM.
4. The enrolled patients are randomly assigned to receive either vitamin D3 or a placebo at the Division of Molecular Epidemiology at JUSM. At IUHW Hospital, the patients are instructed to swallow two capsules per day (containing either supplement or placebo) until the end of the study. Outpatients receive capsules from the CRC.
5. The CRC informs the Division of Molecular Epidemiology at JUSM of the study endpoint by fax.

Outcomes

Primary outcome: relapse-free survival (RFS)

Secondary outcome: overall survival (OS), relapse, all-cause death, cancer-specific death

Safety outcomes: kidney stone, bone fracture, serious adverse events requiring admission. Cancer that appears *de novo* in organs other than the site of the primary cancer after starting supplementation will be included as an adverse event, not as an outcome.

Statistical analysis

Intent to treat, Kaplan-Meier survival curves, Cox hazards models.

Ethics

1. Private information is carefully protected, since human genomes are analyzed.
2. All personal information about the participants is rendered innominate in a linkable fashion at IUHW Hospital.
3. Liability insurance is obtained to compensate patients for side effects of vitamin D or the

placebo.

Funding

This study receives funding from the Department of Surgery, IUHW Hospital, Division of Molecular Epidemiology at JUSM and the Ministry of Education, Culture, Sports, Science and Technology in the Japan-Supported Program for the Strategic Research Foundation at Private Universities

Background

Higher serum vitamin D3: 25(OH)D levels are associated with longer survival. The prognosis is poorer for patients with colorectal (1) and lung (2) cancer accompanied by lower, than higher serum levels of vitamin D, which we also confirmed in colorectal cancer (3).

Randomized controlled trials (RCT) have investigated whether vitamin D plus calcium supplementation can decrease cancer incidence, but none have aimed to improve the prognosis of patients with cancer. Two major RCT have also investigated whether vitamin D and calcium can prevent fracture as a primary outcome and reduce the incidence of cancer as a secondary outcome (4,5). However, whether vitamin D supplementation can improve the survival of patients with cancer has not been investigated in an RCT as far as we can ascertain.

Therefore, we planned the first randomized, double-blind, placebo-controlled trial to clarify whether vitamin D3 supplementation can improve relapse-free survival (RFS) and overall survival (OS) among all patients and subgroups of patients with digestive tract cancers from the esophagus to the rectum after curative surgical tumor resection.

Observational studies have associated serum levels of 25(OH)D (biomarker of vitamin D status) < 20 ng/mL with increased cancer morbidity and mortality (6), particularly in patients with cancers of the digestive system (7), and colorectal cancer (8-10), which we also confirmed (11). In contrast, the International Agency for Research on Cancer (IARC) warned that cancer morbidity and mortality rates might be higher among patients with serum 25(OH)D levels > 40 ng/mL (12), based on two large prospective cohort studies. The Third National Health and Nutritional Examination Survey (NHANES III) argued for caution against the theory that higher vitamin D levels are associated with a better prognosis because overall cancer mortality, especially in digestive cancers, is elevated in patients with higher 25(OH)D levels (13). The Uppsala Longitudinal Study of Adult Men (ULSAM) found that both low and high concentrations of plasma 25(OH)D were associated in a U-shaped fashion with elevated risk of overall and cancer mortality (14). Thus, we will perform analyses of subgroups by serum 25(OH)D cutoff levels of 20 and 40 ng/mL.

Median survival durations of 21.4, 12.1, and 15.6 months are associated with CC, CT, and TT genotypes among Fok*I* SNPs of the vitamin D receptor (VDR) in patients with lung cancer. These findings suggest that SNPs of VDR may significantly impact prognosis (log

rank $P = 0.005$) (15).

SNPs of vitamin D binding protein (DBP) (rs7041 and rs4588) are associated with 25(OH)D (16). Serum 25(OH)D levels are reduced by 25% in homozygous carriers of the rs7041 at-risk T allele ($p<0.0001$) among patients with COPD (10). We therefore plan to add SNPs of DBP to VDR.

We therefore planned to determine relationships between relapse-free survival (RFS) and serum 25(OH)D levels, and SNPs of VDR and DBP in patients with cancers of the gastrointestinal tract (esophageal, gastric, and colon cancers).

Hypothesis

Mechanisms of anti-cancer effects by vitamin D

Serum levels of the active vitamin D precursor, 25(OH)D increase in response to exposure to sunlight or a vitamin D-rich diet or vitamin D supplementation. In contrast, levels of 1,25$(OH)_2$D that is activated in the kidneys remain constant and are least affected by lifestyle. Cancer cells expressing both 1α-hydroxylase and vitamin D receptor (VDR) uptake 25(OH)D and convert it into 1,25$(OH)_2$D, which binds to VDR in cancer-cell nuclei. This signaling influences gene expression, which consequently induces cell proliferation and apoptosis, and inhibits proliferation, angiogenesis, and metastasis.

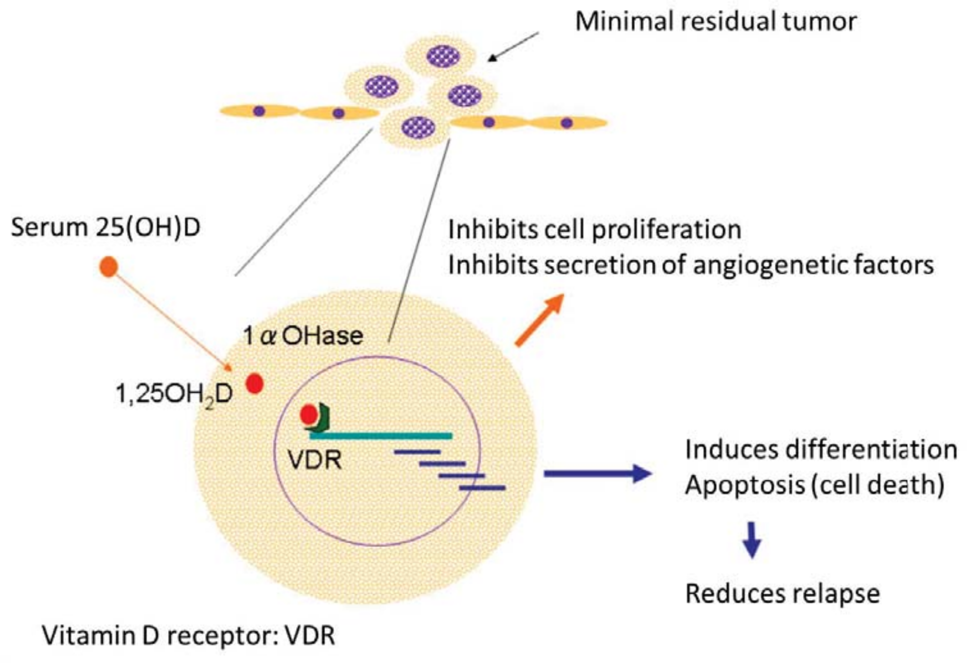

1. Oral vitamin D supplementation causes serum levels of vitamin D (25(OH)D) to increase.
2. Minimal postoperative residual tumors uptake serum 25(OH)D into cancer cells and convert it into active vitamin D (1,25(OH)2D), which binds to nuclear vitamin D receptors within the same cell and influences various cellular functions.
3. As a result, cell proliferation and angiogenesis are suppressed, differentiation and cell death are induced, and minimal residual tumor disappears.
4. Survival can be prolonged by vitamin D compared with a placebo.

Objectives

Specific Aim 1: To determine whether vitamin D3 (2,000 IU) can prevent relapse and death after surgical treatment for cancers patients with the gastrointestinal tract (esophageal gastric and colon) compared with a placebo in total study population and in subgroup stratified by serum 25(OH)D levels with cutoffs at 20 and 40 ng/mL.

Specific Aim 2: To determine relationships between relapse-free survival and serum vitamin D concentrations, SNPs of the vitamin D receptor (VDR) and vitamin D binding protein (DBP) in patients with cancers of the gastrointestinal tract (esophageal, gastric, and colon cancer).

Study design

Specific Aim 1

At the International University of Health and Welfare (IUHW) Hospital, patients with digestive tract cancers from the esophagus to the rectum are randomized in a double-blind, placebo-controlled, parallel-group trial of the effects of vitamin D3 supplements (2,000 IU/day) compared with those of a placebo at an allocation ratio of 3:2 after surgical tumor resection with intent to cure. Relapse-free survival (RFS) and overall survival (OS) are compared between the two groups. Subgroups analyses were stratified according to their having low (< 20 ng/mL), middle, (≥ 20 to ≤ 40 ng/mL) or high (40 ng/mL) 25(OH)D levels.

Specific Aim 2

Relationships between RFS and subgroups of patients with the serum 25(OH)D levels described above and SNPs of VDR and DBP are analyzed.

Research Implementation System

The following medical institutions will collaborate in this trial.

I. Jikei University School of Medicine

Division of Molecular Epidemiology

Urashima M: Conception, design, randomization and data analyses

Akutsu T: Data monitoring

Wada H: Data monitoring

Sakanashi C: SNP analysis

Tago N: SNP analysis

Mafune H: SNP analysis

Suga D: SNP analysis

II. International University of Health and Welfare Hospital（IUHW Hospital）

Department of Surgery

Suzuki Y: Patient entry, treatment, data collection

Ohdaira H: Patient entry, treatment, data collection

Yoshida M: Patient entry, treatment, data collection

Okada S: Pathology

Kitajima M: Critical appraisal of draft

Ohtsuki Y: Clinical Research Coordinator (CRC)

Patients

Inclusion criteria

1. Histopathologically diagnosed epidermal carcinoma of the digestive tract (esophageal, gastric, small intestinal or colorectal mucosa).
2. Clinical stages I to III.
3. Age 30 – 90 years at entry.
4. Diagnosed and first surgery at IUHW Hospital.
5. Not taking vitamin D supplement or active vitamin D.
6. No previous history of urinary tract stones.

Exclusion criteria

1. Tumor that could not be totally resected by surgery.
2. Serious postoperative complications before starting supplementation.
3. Pathological diagnosis other than epidermal carcinoma (malignant lymphoma, sarcoma, etc.).
4. Pathological stage 0 or IV.

Interventions

Per oral supplementation with vitamin D3 or placebo at IUHW Hospital

Enrolled patients are randomly assigned to receive either vitamin D3 supplements (2 × 1,000 IU capsules/day) or placebo (2 capsules/day) starting from the first postoperative assessment as an outpatient until the end of the trial. The two capsules could be taken together or as one each twice daily. The placebo comprised sesame oil, gelatin derived from swine, and glycerin and the active supplement contained the same constituents plus vitamin D3.

Outcome Measures

Primary outcome

1. Relapse-free survival (RFS) is defined as elapsed time from starting supplementation to the earliest date of cancer relapse or death from any cause. Participants who do not relapse and remain alive on the day the trial ends are censored. Survival duration is defined as being from the supplement start day to final outpatient day.

Secondary outcomes

1. Overall survival (OS) defined as elapsed time from the date of starting supplementation to the date of death from any cause. Participants who remain alive on the day the trial ends are censored. Survival duration is defined as being from the supplement start day to the final outpatient day.
2. Relapse: Patients were periodically (1–6 months) examined by CT, MRI, PET and other modalities as needed on an outpatient basis to exclude cancer relapse.
3. All-cause death.
4. Death due to progressive cancer, excluding *de novo* cancer.
5. Death from non-cancer causes such as myocardial infarction and *de novo* cancer progression.

Tertiary outcome

1. De novo cancer appearing in organs other than site of primary cancer after starting supplementation.

Safety outcomes

1. Urinary stone.
2. Hypercalcemia.

3. Bone fracture.
4. Serious events requiring admission.

Flow of participants

1. **Informed consent and registration** at IUHW Hospital

   When a patient is considered eligible, the collaborating surgeon describes the trial purposes etc. to the patients and their families at the hospital outpatient clinic or during admission before surgery and seeks their agreement to participate. Written, informed consent is obtained from each participant. The participant is then assigned an identification number for a study ID and a registration form (**Appendix 3**) with the study ID, age, sex, and key inclusion and exclusion criteria and without personal information is sent by fax from IUHW Hospital to the data monitoring center at JUSM.

2. **Surgical curative resection of tumor and chemotherapy** at IUHW Hospital

   The following are grounds for excluding patients after initial registration:

1. Tumor that could not be totally resected by surgery.
2. Serious postoperative complications before starting supplementation.
3. Pathological diagnosis other than epidermal carcinoma (malignant lymphoma, sarcoma, etc.).
4. Pathological stage 0 or IV.

Chemotherapy

a. Pre- and post-operative chemotherapy is administered to patients with stage II and III esophageal cancer.
b. Post-operative chemotherapy is administered to patients with stage II and III gastric cancer (11)
c. Post-operative chemotherapy is administered to patients with stage III colorectal cancer (12).
d. Local radiation or molecular targeting therapy is combined with chemotherapy for selected patients with relapse.

3. **Clinical information before intervention** at IUHW Hospital

The following information is summarized by the CRC and sent to the data monitoring center at JUSM by fax (**Appendix 3**).

1. Age
2. Sex
3. Diagnosis (e.g., gastric cancer)
4. Stage before operation
5. Pathological stage
6. Pathology
7. Tumor resection: complete resection; microscopically not resected (=edge positive); macroscopic residual tumor remained in the body
8. Sampling: serum for 25(OH)D; blood for genomic DNA extraction; tumor tissue for somatic DNA extraction
9. Anthropometric measurements: height, weight, abdominal circumference, blood pressure
10. Blood tests: Calcium, ALP, parathyroid hormone, total cholesterol, HDL-cholesterol, triglyceride, blood sugar, HbA1c, BUN, Cr

4. **Blood sampling** at IUHW hospital

Blood sampled for serum 25(OH)D measurements and DNA extraction at IUHW Hospital is sent to SRL Inc.

a. **Measurement of serum 25(OH)D levels**

Serum levels of 25(OH)D are measured by radioimmunoassay at SRL Inc. (Hachioji, Tokyo, Japan) before and annually (around the same calendar month) after starting supplementation. Levels for 25(OH)D and residual serum samples are sent to the data monitoring center at JUSM for storage at -80°C for post hoc analysis.

b. **SNP analyses of vitamin D receptor and vitamin D binding protein**

Peripheral blood are sampled from participants at IUHW hospital and sent to SRL Inc., where DNA is extracted. Purified genomic DNA is sent from SRL Inc. to Division of Molecular Epidemiology at JUSM. DNA fragments are amplified by PCR using the forward/reverse primers listed below and the conditions described in **Appendix 1** (13). The SNPs are determined by direct sequencing. Samples are stored at -80°C.

SNPs

a. Vitamin D receptor (VDR): Fok*I*, rs10735810; Bsm*I*, rs1544410; CDX2, rs11568820; Apa*I*, rs7976091; Taq*I*, rs731236
b. Vitamin D binding protein (DBP): DBP1, rs7041; DBP2, rs4588

c. DNA extraction from tumor tissue

Tumor samples obtained during surgery at IUHW Hospital are sent to SRL Inc., where DNA is extracted. Purified somatic DNA is sent from SRL Inc. to the data monitoring center at JUSM and stored at -80°C for future studies.

5. Randomization and double blinding at JUSM

a. Supplementation

Both vitamin D3 and placebo (Zenyaku Pharmaceutical Co., Ltd., Tokyo, Japan) are prepared as soft capsules containing either 1,000 IU of vitamin D3 or a placebo. All capsules are identical in appearance and taste, and packaged in lots of 366 capsules in identical brown glass bottles. Both supplements are purchasable from Zenyaku Pharmaceutical Co., Ltd., Tokyo, Japan.

b. Randomization

M.U. at the data monitoring center has no clinical involvement in this trial. M.U. generates random numbers from 1 to 10 using a computer, assigns permutated blocks of five to fit in a 3:2 ratio (**Appendix 2**), and creates a correspondence table to link the study ID to either vitamin D3 or placebo.

c. Double-blinding

An administrative staff member and M.U. label each bottle with the study ID and confirm the ID number with the correspondence table. Bottles labeled in this manner are periodically sent from the data monitoring center to IUHW Hospital. Staff at the data monitoring center have no contact with participants at IUHW Hospital. Thus, the participants in this trial and all the staff including surgeons who assess relapse at IUHW Hospital are completely blinded to which patients received supplement or placebo.

6. Compliance with supplementation at IUHW Hospital

1. Patients are questioned about compliance at every visit.
2. Levels of 25(OH)D are annually measured in blood samples to determine changes in the vitamin D and placebo groups.

7. **Reports of relapse and death** at IUHW Hospital

Reports of relapse or death are prepared at IUHW Hospital and sent to the data monitoring center at JUSM by fax (**Appendix 3**).

a. Relapse

In addition to date of relapse, the surgeon in charge or the CRC describes in detail why a patient is diagnosed as having relapsed from MRI findings, and other findings, such as local recurrence, lymph node metastasis, distant metastasis, or peritoneal dissemination.

b. Death

In addition to the date of death, the surgeon in charge or the CRC details causes of death, such as cancer progression, to determine death from cancer or non-cancer.

c. **Censor**

Participants are censored in terms of RFS if they are relapse-free or alive at the end of the trial.

Participants are censored in terms of OS if they have not died of any cause at the end of the trial. Survival is defined as elapsed time between the dates of starting supplementation and the final visit to the outpatient clinic.

8. **Reports of safety outcomes** at IUHW Hospital

Reports of safety outcomes (urinary stone, hypercalcemia, bone fracture, severe adverse events requiring admission, double cancer, and others) prepared at IUHW hospital are sent to the data monitoring center at JUSM by fax (**Appendix 3**).

If medically considered difficult to continue taking supplements or if a participant desires to stop taking supplements, the surgeon in charge can decide to stop supplementation.

9. **Follow-up** at IUHW hospital

At least once per year, the CRC reports the date and status of participants at their final visit to the outpatient clinic to personnel at the data management center to ensure that all participants are followed up.

Statistical analysis

1. Sample size

Target sample size, 400 patients. Vitamin D group, n = 240; placebo group, n = 160.

2. Sample size calculation

We postulated that the 5-year RFS would be 75% and 62% in the vitamin D and placebo groups, respectively, with a type I error (two-sided) of 5% and a power of 80%, assuming a 1% loss to follow-up. Therefore, we calculated that 400 patients with digestive tract cancers divided in a 3:2 ratio (Vitamin D group, n = 240; placebo group, n = 160) would be sufficient to detect a significant difference.

Log of sample size calculation using Stata:

```
st power log rank 0.62 0.75, n ratio(1.5) wd prob (0.01)


Estimated sample sizes for two-sample comparison of survivor functions
Log-rank test, Freedman method
Ho: S1(t) = S2(t)

Input parameters:

      alpha =    0.0500    (two sided)
         s1 = 0.6200
         s2 = 0.7500
    h ratio = 0.6018
      power =    0.8000
         p1 = 0.4000
 withdrawal = 1.00%

Estimated number of events and sample sizes:

          E = 120
          N = 400
         N1 = 160
         N2 = 240
```

3. **Study period**

Fifty patients each with gastric and colorectal cancers and 10 with esophageal cancer are treated annually at the Department of Surgery, IUHW Hospital. We assumed that 80 patients per year could participate in this trial. Therefore, the accrual period will be 5 years. After enrolling 400 patients, enrollment will finish, and the patients will be followed up for two more years. Thus, the total length of planned study is 7 years.

4. **Interim analysis**

Annual interim analyses are planned after entry of 200 patients. The P value for significance at the interim analysis is <0.001 according to Peto stopping boundaries (14).

5. **Planned methods of analysis**

    a. Changes of 25(OH)D levels will be analyzed using Wilcoxon signed-rank tests.
    b. Comparisons of patients' characteristics between vitamin D and placebo will be analyzed using Student t-tests and Mann-Whitney tests for continuous variables with normal and non-normal distribution, respectively. Dichotomous outcomes are calculated using chi-square tests.
    c. Kaplan-Meier survival curves will be created on an intent-to-treat analysis.
    d. Cox proportional hazard model will be used to determine hazard ratio (HR) and 95% confidence intervals (95%CI) of RFS and OS.
    e. Relapse and safety outcomes will be evaluates using risk ratio (RR).
    f. All reported P values will be two-sided.
    g. Values with $P < 0.05$ will be considered statistically significant.
    h. All data will be statistically analyzed using Stata 14.0 (StataCorp LP., College Station, TX, USA).

6. **Subgroup analyses**

To clarify whether vitamin D supplementation significantly affects the subgroups listed below, P for interaction ($P_{interaction}$) is computed by creating multiplicative variables. The results of these analyses are not corrected for multiple comparisons.

Subgroup analyses

i. 25(OH)D values: low, <20 ng/mL; middle, ≥20 to ≤40 ng/mL; high, >40 ng/mL.
ii. VDR: Fok*I*, Bsm*I*, CDX2, Taq*I*, Apa*I*
iii. DBP1, DBP2

Safety

Serious adverse events or side effects caused by vitamin D3 supplements are rare.

All capsules contain sesame oil, gelatin derived from swine, and glycerin; thus, participants in the vitamin D and placebo groups who might be sensitive to any of these components could develop nausea and vomiting.

The Japanese Ministry of Health, Labor and Welfare suggests that the safe range of vitamin D3 supplementation is between 200 and 2,000 IU/day for healthy adults.

Early withdrawal

a. Unknown serious adverse events and side effects of vitamin D.
b. Frequent known side effects such as nausea and vomiting.
c. Frequent theoretically plausible side effects such as hypercalcemia.

Compensation

Mitsui Sumitomo Insurance Fire provides liability insurance for the principal investigator, to compensate for costs of treating disability as a result of side effects caused by taking the trial supplement.

The maximum amount of compensation is 100,000,000 yen per person and 300,000,000 yen per trial.

Consideration concerning the protection of human rights and privacy

1. Written informed consent is obtained after sufficient explanation.
2. Participants can withdraw from the trial after providing written, informed consent.
3. Withdrawal is not considered a disadvantage for participants.
4. Private information is exchanged with study ID at IUHW Hospital; therefore, private information cannot leak from IUHW Hospital (linkable anonymizing).
5. Private information is not collected at Division of Molecular Epidemiology, JUSM.
6. Contact point regarding study ID.

Disclosure of genetic information

Information disclosure regarding vitamin D related SNPs can be provided by the surgeon in charge at IUHW Hospital if a participant requests such.

Publication of research results

We aim to publish the results of this trial in a scientific journal or present them at academic conferences. Personal information will be carefully concealed when we present the results.

Research funds

The present study will receive funding from the Department of Surgery, IUHW Hospital, Division of Molecular Epidemiology, the Ministry of Education, Culture, Sports, Science and Technology in the Japan-Supported Program for the Strategic Research Foundation at Private Universities

Attribution of intellectual property rights

If intellectual property rights such as patent rights become relevant, such rights will be attributable to the investigators.

References

1. Ng K, Meyerhardt JA, Wu K, Feskanich D, Hollis BW, Giovannucci EL, Fuchs CS. Circulating 25-hydroxyvitamin d levels and survival in patients with colorectal cancer. J Clin Oncol. 2008 Jun 20;26(18):2984-91.
2. Zhou W, Heist RS, Liu G, Asomaning K, Neuberg DS, Hollis BW, Wain JC, Lynch TJ, Giovannucci E, Su L, Christiani DC. Circulating 25-hydroxyvitamin D levels predict survival in early-stage non-small-cell lung cancer patients. J Clin Oncol. 2007 Feb 10;25(5):479-85.
3. Mezawa H, Sugiura T, Watanabe M, Norizoe C, Takahashi D, Shimojima A, Tamez S, Tsutsumi Y, Yanaga K, Urashima M. Serum vitamin D levels and survival of patients with colorectal cancer: post-hoc analysis of a prospective cohort study. BMC Cancer. 2010 Jul 2;10:347. doi: 10.1186/1471-2407-10-347.
4. Wactawski-Wende J, Kotchen JM, Anderson GL, Assaf AR, Brunner RL, O'Sullivan MJ, Margolis KL, Ockene JK, Phillips L, Pottern L, Prentice RL, Robbins J, Rohan TE, Sarto GE, Sharma S, Stefanick ML, Van Horn L, Wallace RB, Whitlock E, Bassford T, Beresford SA, Black HR, Bonds DE, Brzyski RG, Caan B, Chlebowski RT, Cochrane B, Garland C, Gass M, Hays J, Heiss G, Hendrix SL, Howard BV, Hsia J, Hubbell FA, Jackson RD, Johnson KC, Judd H, Kooperberg CL, Kuller LH, LaCroix AZ, Lane DS, Langer RD, Lasser NL, Lewis CE, Limacher MC, Manson JE; Women's Health Initiative Investigators. Calcium plus vitamin D supplementation and the risk of colorectal cancer. N Engl J Med. 2006 Feb 16;354(7):684-96.
5. Lappe JM, Travers-Gustafson D, Davies KM, Recker RR, Heaney RP. Vitamin D and calcium supplementation reduces cancer risk: results of a randomized trial. Am J Clin Nutr. 2007 Jun;85(6):1586-91.
6. Holick MF. Vitamin D deficiency. N Engl J Med. 2007 Jul 19;357(3):266-81.
7. Giovannucci E, Liu Y, Rimm EB, Hollis BW, Fuchs CS, Stampfer MJ, Willett WC. Prospective study of predictors of vitamin D status and cancer incidence and mortality in men. J Natl Cancer Inst. 2006 Apr 5;98(7):451-9.
8. Garland CF, Comstock GW, Garland FC, Helsing KJ, Shaw EK, Gorham ED. Serum 25-hydroxyvitamin D and colon cancer: eight-year prospective study. Lancet. 1989 Nov 18;2(8673):1176-8.
9. Ng K, Meyerhardt JA, Wu K, Feskanich D, Hollis BW, Giovannucci EL, Fuchs CS. Circulating 25-hydroxyvitamin d levels and survival in patients with colorectal cancer. J Clin Oncol. 2008 Jun 20;26(18):2984-91.
10. Ng K, Wolpin BM, Meyerhardt JA, Wu K, Chan AT, Hollis BW, Giovannucci EL, Stampfer

MJ, Willett WC, Fuchs CS. Prospective study of predictors of vitamin D status and survival in patients with colorectal cancer. Br J Cancer. 2009 Sep 15;101(6):916-23.

11. Mezawa H, Sugiura T, Watanabe M, Norizoe C, Takahashi D, Shimojima A, Tamez S, Tsutsumi Y, Yanaga K, Urashima M. Serum vitamin D levels and survival of patients with colorectal cancer: post-hoc analysis of a prospective cohort study. BMC Cancer. 2010 Jul 2;10:347. doi: 10.1186/1471-2407-10-347.
12. Freedman DM1, Looker AC, Abnet CC, Linet MS, Graubard BI. Serum 25-hydroxyvitamin D and cancer mortality in the NHANES III study (1988-2006). Cancer Res. 2010 Nov 1;70(21):8587-97.
13. Michaëlsson K, Baron JA, Snellman G, Gedeborg R, Byberg L, Sundström J, Berglund L, Arnlöv J, Hellman P, Blomhoff R, Wolk A, Garmo H, Holmberg L, Melhus H. Plasma vitamin D and mortality in older men: a community-based prospective cohort study. Am J Clin Nutr. 2010 Oct;92(4):841-8.
14. https://www.iarc.fr/en/publications/pdfs-online/wrk/wrk5/Report_VitD.pdf
15. Heist RS, Zhou W, Wang Z, Liu G, Neuberg D, Su L, Asomaning K, Hollis BW, Lynch TJ, Wain JC, Giovannucci E, Christiani DC. Circulating 25-hydroxyvitamin D, VDR polymorphisms, and survival in advanced non-small-cell lung cancer. J Clin Oncol. 2008 Dec 1;26(34):5596-602.
16. Janssens W1, Bouillon R, Claes B, Carremans C, Lehouck A, Buysschaert I, Coolen J, Mathieu C, Decramer M, Lambrechts D. Vitamin D deficiency is highly prevalent in COPD and correlates with variants in the vitamin D-binding gene. Thorax. 2010 Mar;65(3):215-20.

Appendix 1

Table 1. Primers and PCR conditions

	primers	denaturation	cycles	Annealing and extension	Stopping reaction
Fok*I*	5’-ctccgaaggcactgtgctcaggcct/ atggaaacaccttgcttcttctccctc-3’	98ºC for 1 min	30 cycles at 98ºC for 10 s	68ºC for 4 min	16ºC
Cdx2	5’-gggaaggagggagggaggaaggaagg/ agctgtagcaatgaaagcaaacc-3	95ºC for 3 min	30 cycles at 95ºC for 90 s	59ºC for 90 s, then at 72ºC for 2 min	16ºC
BsmI	5’-gctgagggccagctgggcaacctgaa/ aaccagcgggaagaggtcaaggg-3’	94°C for 3 min	35 cycles at 94°C for 20 s	62°C for 40 s, extension at 72°C for 1 min, then final extension at 72°C for 6 min	16°C
ApaI/ TaqI	5’-agagcatggacagggagcaaggccaggcag/gcgcaggtcggctagcttctggatcatc-3’	94°C for 10 min	35 cycles at 93°C for 45 s	66°C for 30 s, extension at 72°C 45 s, then final extension at 72°C for 10 min	16°C
DBP1/ DBP2	5’-cgaagaggcatgtttcactttctgatctca-3’/ 5’-gccattatgtgacaggcttttcctggtg-3’	94ºC for 10 min	25 cycles at 94ºC for 45 sec	55ºC for 30 sec, 72ºC for 1 min	16°C

Appendix 2

Table 2. Permutated blocks of five

1	2	3	4	5	6	7	8	9	10
A	A	A	A	A	A	P	P	P	P
A	A	A	P	P	P	A	A	A	P
A	P	P	A	A	P	A	A	P	A
P	A	P	A	P	A	A	P	A	A
P	P	A	P	A	A	P	A	A	A

Appendix 3

Registration form

If you obtained written informed consent from the participant, please send the following information by FAX.

Registration date：________year、________month、________day
Person who fills out a form：

____

Age _____________
Sex ：male ☐；female ☐

Inclusion criteria：If it is yes, the check the boxes. ☑

☐ Age 30≤ < 90 years of age

☐ First operation for cancer of gastrointestinal tract at International University of Health and Welfare hospital

☐ Obtained informed consent

☐ Any one of following exclusion criteria do not apply

Exclusion criteria：

☐ Already taking vitamin D supplement or 1, 25 vitamin D

☐ History of kidney stone

☐ Other difficulties judged by the surgeon in charge

Special Notes : In past history or family history, if there are specific underlying diseases, e.g., diabetes, please specify in detail following space.

Clinical Information

If you obtained all of available information, please send the following information by FAX.

Date : ________year、________month、________day

Person : _____________________________________。

Date of start supplementation : ________ 年、________ 月、________ 日

Diagnosis ____________________ （e.g., gastric cancer）

Pathological stage _______________

Pathology ________________

Tumor resection: ☐ complete resection; ☐ microscopically not resected (=edge positive); ☐ macroscopic residual tumor remained in the body

Sampling: ☐ serum for 25(OH)D; ☐ blood for genomic DNA extraction; ☐ tumor tissue for somatic DNA extraction

Body height__________、body weight__________、blood pressure ________/________

Abdominal circumference__________

Blood examination

Select timing of blood sampling : fasting; within 2 hours after meal; within 4 hours after meal

Calcium __________、ALP __________

PTH ________________

Total Cholesterol __________、HDL Cholesterol__________

Triglyceride __________、

Blood sugar __________、HbA1c __________

BUN__________、Cr __________、

Special Notes

Report at relapse

When the participant is confirmed relapse, please send us following information.

Writing date : ________year、________month、________day
Writing personnel : _________________________________

relapse : date ________year、________month、________day
place（organ）

Please write down detailed in relapse.

Report at decease

When the participant has deceased, please send us following information.

Writing date：________year、________month、________day
Writing personnel： _________________________________

Deceased date ________year、________month、________day
place（organ）
cause ：please check either one
□ primary cancer death (death caused by double cancer is not included cancer death, but include non-primary cancer death)
□ non-primary cancer death: please write down following space.
Example: year 2012 March 1st, tumor relapse at primary lesion was confirmed by MRI. Chemotherapy was applied, but the tumor continued to grow and did not reach remission, and finally died at year 2012 June 26th.

Annual report during follow-up

Every year, please send us annual information.

Writing date : ________year、________month、________day
Writing personnel : _________________________________

If participant stop taking the supplements, please select a reason from followings;

☐ Most of duration, i.e., more than 11 months, participant took the supplements during one year.

☐ Less than 11 months, the patient took the supplements.
Specify the duration of period : _____________

Additional treatment other than surgery

□ chemotherapy、□ radiation local therapy, ☐ other therapy; specify following spece;

Body height__________、body weight__________、blood pressure ________/________
Abdominal circumference__________

Blood examination

Select timing of blood sampling : fasting; within 2 hours after meal; within 4 hours after meal

Calcium __________、ALP __________
PTH ________________
Total Cholesterol __________、HDL Cholesterol__________
Triglyceride __________、
Blood sugar __________、HbA1c __________
BUN__________、Cr __________、

Special Notes

Adverse events

When the participant was needed admission due to adverse events except cancer relapse and cannot deny possibility of causal relation with supplementation, please send us following information by FAX.

Writing date：________year、________month、________day
Writing personnel：____________________________________

Adverse event date ________year、________month、________day
Known：**digestive tract signs**（nausea, vomiting, diarrhea, etc）
□ possible causal relation □ causal relation □not for sure

：**hypercalcemia**
□ possible causal relation □ causal relation □not for sure
：**urine stone**
□ possible causal relation □ causal relation □not for sure

Unknown
Severe：needing admission
Please specify in detail

Stop follow-up

Please select one from followings;

☐ Patient wants to stop.

☐ Patient does not come outpatient clinic and cannot contact any more.

☐ By moving etc., patient has difficulty in coming outpatient clinic.

☐ Other reasons：Please specify following space;

Final outpatient visit： ________year________month________day

State at final visit

☐ No relapse

☐ On relapse

Summary of changes to the Trial Protocol and Statistical Analysis Plan (SAP)

ver.1 : 2008.12.25

ver.2 : 2009.10.8 revision

ver.3 : 2012.12.18 revision

Dose of vitamin D supplement

Dose of vitamin D per day was increased from 1,200IU/day to 2,000IU/day before starting the trial at 2009.10.8.

Sample size

Since this is the first trial, it was difficult to predict survival rate in vitamin D group and placebo group. Thus, sample size was blank at 2008.12.25, but fixed as 400 before starting the trial at 2009.10.8.

Subgroup analysis

We decided to perform subgroup analyses stratified by serum levels of 25(OH)D at 20ng/ml and 40ng/ml, after starting this trial and before first midterm analysis, at 2012.12.18.

Polymorphisms of vitamin D binding protein

In addition to SNPs analyses of the vitamin D receptor (VDR), vitamin D binding protein (DBP) was added at 2012.12.8.

Double (triple) cancer incidence

De novo cancer appearing in organs other than site of primary cancer after starting supplementation was first included in severe adverse events. However, separated from secondary and safety outcomes and inserted as tertiary outcome at 2012.12.8.
