# Supplementary material for: Effect of Vitamin D Supplements on Relapse or Death in a p53-Immunoreactive Subgroup With Digestive Tract Cancer: Post Hoc Analysis of the AMATERASU Randomized Clinical Trial
Source: JAMA Netw Open. 2023 Aug 22;6(8):e2328886. doi: 10.1001/jamanetworkopen.2023.28886 (PMC10445201; doi:10.1001/jamanetworkopen.2023.28886)
Supplement: Supplement 2. — eTable. Characteristics of Patients Who Were Anti-p53 Antibody Positive by Intervention [file jamanetwopen-e2328886-s002.pdf]

Supplemental Online Content

Kanno K, Akutsu T, Ohdaira H, Suzuki Y, Urashima M. Effect of vitamin D supplements on relapse or death in a p53-immunoreactive subgroup with digestive tract cancer: post hoc analysis of the AMATERASU randomized clinical trial. *JAMA Netw Open*. 2023;6(8):e2328886. doi:10.1001/jamanetworkopen.2023.28886

eTable. Characteristics of Patients Who Were Anti-p53 Antibody Positive by Intervention

This supplemental material has been provided by the authors to give readers additional information about their work.

© 2023 Kanno K et al. *JAMA Network Open.*

eTable 1. Characteristics of p53-Ab (+) patients stratified by intervention

	Intervention	
	Vitamin D<br>N=86	Placebo<br>N=56
25(OH)D[a], n (%)		
Low: <20 ng/mL	38 (45)	18 (32)
Middle: ≥20 and ≤40 ng/mL	45 (53)	37 (66)
High: >40 ng/mL	2 (2)	1 (2)
25(OH)D[a] serum level, ng/mL		
Median<br>IQR (25%-75%)	20<br>(16-25)	22<br>(16-27.5)
Sex, n (%)		
Male	60 (70)	36 (64)
Female	26 (30)	20 (36)
Age quartile, n (%)		
Q1, 35-59 years	21 (24)	19 (34)
Q2, 60-65 years	14 (16)	13 (23)
Q3, 66-73 years	18 (21)	16 (29)
Q4, 74-90 years	33 (38)	8 (14)
Body mass index quartile, n (%)		
Q1, 15.0-19.7 kg/m^2	23 (27)	14 (25)
Q2, 19.8-21.8 kg/m^2	22 (26)	14 (25)
Q3, 21.9-23.7 kg/m^2	20 (24)	16 (29)
Q4, 23.8-37.3 kg/m^2	20 (24)	12 (21)
History of other cancers, n (%)	3 (3)	2 (4)
Comorbidities, n (%)		
Hypertension	28 (33)	18 (32)
Diabetes mellitus	19 (22)	6 (11)
Endocrine disease	4 (5)	2 (4)
Cardiovascular disease	6 (7)	0 (0)
Chronic kidney disease	4 (5)	1 (2)
Asthma	1 (1)	0 (0)
Orthopedic disease	0 (0)	0 (0)
Site of cancer, n (%)		
Esophageal	13 (15)	8 (14)

© 2023 Kanno K et al. *JAMA Network Open.*

Stomach	27 (31)	25 (45)
Small bowel	0 (0)	0 (0)
Colorectal	46 (53)	23 (41)
Stage, n (%)		
I	30 (35)	21 (38)
II	28 (33)	16 (29)
III	28 (33)	19 (34)
Pathology		
Adenocarcinoma, n (%)	72 (84)	48 (86)
Squamous cell carcinoma, n (%)	13 (15)	8 (14)
Other, n (%)	1 (1)	0 (0)
Adjuvant chemotherapy, n (%)	35 (41)	24 (43)

[a] 25-hydroxy vitamin D

© 2023 Kanno K et al. *JAMA Network Open.*
