# Supplementary material for: Effect of Vitamin D Supplements on Relapse or Death in a p53-Immunoreactive Subgroup With Digestive Tract Cancer: Post Hoc Analysis of the AMATERASU Randomized Clinical Trial
Source: JAMA Netw Open. 2023 Aug 22;6(8):e2328886. doi: 10.1001/jamanetworkopen.2023.28886 (PMC10445201; doi:10.1001/jamanetworkopen.2023.28886)
Supplement: Supplement 3. — Data Sharing Statement [file jamanetwopen-e2328886-s003.pdf]

Data Sharing Statement

Kanno. Effect of Vitamin D Supplements on Relapse or Death in a p53-Immunoreactive Subgroup With Digestive Tract Cancer: Post Hoc Analysis of the AMATERASU Randomized Clinical Trial. *JAMA Netw Open*. Published online August 15, 2023.
doi:10.1001/jamanetworkopen.2023.28886

Data
Data available: Yes
Data types: Deidentified participant data
How to access data: The principal investigator (urashima@jikei.ac.jo) had full access to all the data in the study and takes responsibility for the integrity of the data and the accuracy of the data analysis.
When available: With publication

Supporting Documents
Document types: None

Additional Information
Who can access the data: The principal investigator (urashima@jikei.ac.jo) had full access to all the data in the study and takes responsibility for the integrity of the data and the accuracy of the data analysis.
Types of analyses: for review and meta-analysis
Mechanisms of data availability: after approval of a proposal
